# A Review on the Degradation of Pollutants by Fenton-Like Systems Based on Zero-Valent Iron and Persulfate: Effects of Reduction Potentials, pH, and Anions Occurring in Waste Waters

**DOI:** 10.3390/molecules26154584

**Published:** 2021-07-29

**Authors:** Naveed Ahmed, Davide Vione, Luca Rivoira, Luca Carena, Michele Castiglioni, Maria Concetta Bruzzoniti

**Affiliations:** Department of Chemistry, University of Turin, Via Pietro Giuria 5, 10125 Turin, Italy; luca.rivoira@unito.it (L.R.); luca.carena@unito.it (L.C.); michele.castiglioni@unito.it (M.C.)

**Keywords:** Fenton chemistry, zero valent iron (ZVI), waste water treatment, water matrices, pH

## Abstract

Among the advanced oxidation processes (AOPs), the Fenton reaction has attracted much attention in recent years for the treatment of water and wastewater. This review provides insight into a particular variant of the process, where soluble Fe(II) salts are replaced by zero-valent iron (ZVI), and hydrogen peroxide (H_2_O_2_) is replaced by persulfate (S_2_O_8_^2−^). Heterogeneous Fenton with ZVI has the advantage of minimizing a major problem found with homogeneous Fenton. Indeed, the precipitation of Fe(III) at pH > 4 interferes with the recycling of Fe species and inhibits oxidation in homogeneous Fenton; in contrast, suspended ZVI as iron source is less sensitive to the increase of pH. Moreover, persulfate favors the production of sulfate radicals (SO_4_^•−^) that are more selective towards pollutant degradation, compared to the hydroxyl radicals (^•^OH) produced in classic, H_2_O_2_-based Fenton. Higher selectivity means that degradation of SO_4_^•−^-reactive contaminants is less affected by interfering agents typically found in wastewater; however, the ability of SO_4_^•^^−^ to oxidize H_2_O/OH^−^ to ^•^OH makes it difficult to obtain conditions where SO_4_^•−^ is the only reactive species. Research results have shown that ZVI-Fenton with persulfate works best at acidic pH, but it is often possible to get reasonable degradation at pH values that are not too far from neutrality. Moreover, inorganic ions that are very common in water and wastewater (Cl^−^, HCO_3_^−^, CO_3_^2−^, NO_3_^−^, NO_2_^−^) can sometimes inhibit degradation by scavenging SO_4_^•−^ and/or ^•^OH, but in other cases they even enhance the process. Therefore, ZVI-Fenton with persulfate might perform unexpectedly well in some saline waters, although the possible formation of harmful by-products upon oxidation of the anions cannot be ruled out.

## 1. Introduction

Advanced oxidation processes (AOPs) are emerging alternative methods for the effective removal of organic and inorganic wastewater pollutants with high stability and/or low biodegradability. These processes are mainly based on the generation of highly reactive radical species, among which the most common one is the hydroxyl radical, ^•^OH [1]. In homogenous systems, radical species are being generated through several ways including electron transfer during activation of oxidants by transition metals, photolysis, thermolysis, and sonolysis [2]. The most conventional homogenous AOP is the Fenton process, in which H_2_O_2_ is activated by ferrous ions to produce ^•^OH [3]. The AOPs operate at ambient temperature and pressure and are often able to induce complete degradation of pollutants into non-toxic products like CO_2_, H_2_O, and inorganic salts [4].

The Fenton process is being widely used as a common AOP for the decomposition of organic and inorganic pollutants in wastewater [5]. As a robust, convenient, and easy method [6], the Fenton process can be used either to lower toxicity of wastewater or to decontaminate wastewaters to allow for their drainage to water bodies [5,7]. In the traditional homogeneous Fenton process, strong oxidants (^•^OH and/or reactive high-valence Fe species, such as ferryl, FeO^2+^) are generated by reaction of H_2_O_2_ with soluble iron ions (Fe^2+^) that act as catalysts in acidic conditions [8]. The use of soluble Fe salts (Fe^2+^) in traditional Fenton gives the highest efficiency if we ignore the mass transfer limitations between active reagents [9]. The main drawback of the classical Fenton process is Fe(III) precipitation due to both catalyst (Fe^2+^) oxidation and pH adjustment, which produces a sludge waste [5,10,11]. Indeed, Fe^2+^ remains dissolved even at neutral pH but Fe^3+^ disappears at pH ≥ 4 while forming ferric hydroxides sludge. Therefore, severe acidic conditions (pH < 4) are required to maintain and carry out the process for practical applications, which demand high cost of reagents to acidify the effluents before treatment, and afterward neutralizing them before drainage [12].

To overcome the above-mentioned drawbacks, in the past decades many approaches have been made to proceed with better efficacy while using heterogeneous Fenton and Fenton-like systems. In heterogeneous Fenton reaction, different poorly soluble iron compounds (Fe_2_O_3_, Fe_3_O_4_, FeO, FeS_2_, etc.) are added as catalysts with H_2_O_2_ to decontaminate or degrade pollutants in aqueous solution [4,5,9,13]. Other alternatives to increase the efficiency of the Fenton reaction are to use supports for iron oxides or include Fe oxides into composite porous materials (clay, zeolites, activated carbon, polymers and multiwalled carbon nanotubes, Nafion films, pumice particles, ashes, and aluminates) [4,5,14,15,16,17]. The overall performance of the Fenton reaction may even be increased in such conditions because the support adsorbs the pollutant molecule and, in some cases, can also facilitate different pathways that generate radicals for pollutants degradation [5,18,19,20].

In heterogeneous Fenton-like systems, the generation of free radicals and/or other oxidizing species is achieved by using the heterogeneous catalyst together with ultrasound energy, magnetic field, ultraviolet radiations, microwave radiation, or a combination of them [5,21].

Zero-valent iron (ZVI) has attracted much attention by researchers among the catalysts used for Fenton-like or heterogeneous Fenton processes. ZVI is a promising material to be used for water and wastewater treatments, due to its eco-friendliness [5,22], cost-effectiveness [23], non-toxicity, and ability to transform different pollutants such as halogenated compounds [5,24,25], nitrate [26,27], heavy metals [5,26,28,29,30,31], phosphate [32], arsenic [33,34,35,36], phenol [37,38,39], polycyclic aromatic hydrocarbons [40,41], and dyes [7,42]. ZVI can be used either alone by exploiting its reductive (electron-donor) properties [39], or in combination with H_2_O_2_ in a heterogeneous Fenton reaction with production of oxidizing species and especially ^•^OH [42]. Last but not least, recent advances have focused on the combined use of ZVI with persulfate (S_2_O_8_^2−^) or peroxymonosulfate (SO_5_^2−^) to obtain a heterogeneous Fenton-like process, where the main aim is to generate the sulfate radical (SO_4_^•−^) as an alternative oxidizing agent to ^•^OH. Compared to ^•^OH, SO_4_^•−^ is a bit less reactive but it is more selective [43,44]. This means that, in well-designed systems, the SO_4_^•−^-based processes can achieve pollutant degradation with lesser interference from water components when compared to the ^•^OH-based techniques (vide infra) [45].

Many water parameters have the potential to affect the performance of the ZVI-Fenton and Fenton-like systems towards pollutant degradation. They include, among others, solution chemistry, pH, concentration of reagents, and involvement of different anions [5,11,46,47]. One of the most crucial operating parameters among all others is pH, which controls the chemistry, the capacity to generate radicals, the catalyst behavior and the overall efficiency of virtually any Fenton process [5,48,49].

This review addresses the effects of pH and of interfering ions (chloride, carbonate, bicarbonate and nitrate/nitrite, as important components of water and wastewater) on the Fenton-like process based on ZVI and persulfate (ZVI/PS), which has not been reviewed so far [3,5,13,50]. Furthermore, the formation of secondary pollutants is here discussed. We decided to focus on ZVI/PS instead of ZVI/peroxymonosulfate, which is also an effective technique, because ZVI/PS has been the object of more numerous studies that covered a wider range of conditions. Moreover, by focusing on PS it was also easier to compare the results of different studies.

ZVI/PS is a promising process, which may combine the advantages of heterogeneous Fenton with the selectivity of SO_4_^•−^. However, although the replacement of H_2_O_2_ with persulfate can reduce interferences by water components, it does not totally eliminate them. Moreover, pH still plays a key role because, in addition to affecting the overall Fenton efficiency, it also influences the reaction pathways by favoring the generation of ^•^OH vs. SO_4_^•−^ as the main reactive species, as explored in the next section.

## 2. Reactive Species: ^•^OH vs. SO_4_^•−^ vs. ^•^OH + SO_4_^•−^

One of the main features of the hydroxyl radical is its high reactivity towards most compounds that occur in water and wastewater [51]. On the one side, this warrants the possibility to degrade a wide array of contaminants in the framework of water treatment. At the same time, however, ^•^OH can be scavenged by many constituents of natural water and wastewater, such as organic matter and some inorganic anions (e.g., Cl^−^, NO_2_^−^, HCO_3_^−^, and CO_3_^2−^). Natural water constituents do not necessarily require oxidation for water to be properly decontaminated, thus their ability to scavenge ^•^OH will mostly inhibit the degradation of pollutants [52]. The consequence of ^•^OH scavenging by organic matter and inorganic anions, which act as interfering agents, is thus increased costs and/or a decrease in process efficiency [51].

**Figure 1:**
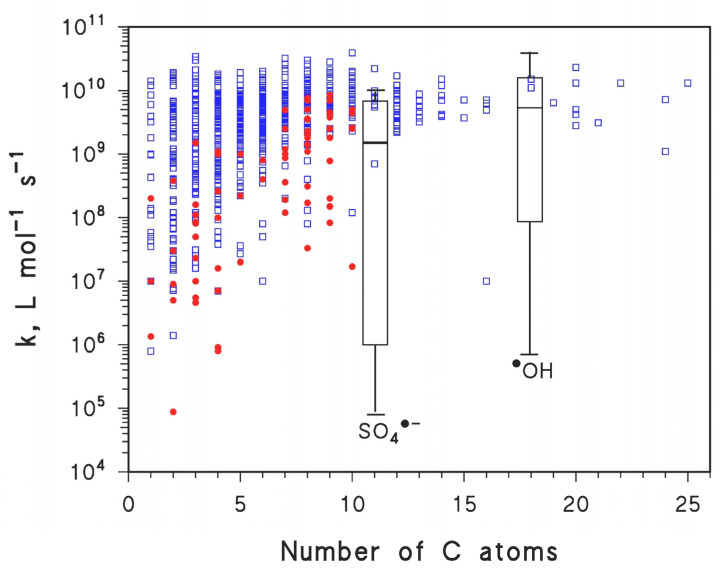
Second-order reaction rate constants (*k*) of organic compounds with SO_4_^•−^ (•, *n* = 119) and with ^•^OH (□, *n* = 713), as a function of the number of carbon atoms (on average, compounds with more carbons are more reactive, which for instance justifies the use of a group contribution method to predict ^•^OH reactivity) [43,44]. In the summarizing diagrams, boxes represent the 5th, 50th (median), and 95th percentiles of the distribution, while the whiskers show the extreme values.

A possible solution to the above problem is to look for alternative reactive species, which should be more selective than ^•^OH but, at the same time, ensure elevated reactivity and oxidizing power. The sulfate radical, SO_4_^•−^, has interesting features because it has similar or even higher reduction potential compared to ^•^OH, but at the same time it is more selective. Figure 1 reports the available literature data about the second-order reaction rate constants between the two radical species (^•^OH, *n* = 713, and SO_4_^•−^, *n* = 119) and organic compounds, here indicated by the respective number of C atoms [43,44]. The comparison suggests that, on average, ^•^OH is more reactive compared to SO_4_^•−^.

Moreover, although the highest SO_4_^•−^ reaction rate constants approach (and those of ^•^OH slightly surpass) the value of 10^10^ L mol^−1^ s^−1^, which is near the diffusion-control limit in aqueous solution [43], the lowest rate constant values reach 10^6^ L mol^−1^ s^−1^ in the case of ^•^OH and 10^5^ L mol^−1^ s^−1^ in the case of SO_4_^•−^.

Overall, the reaction rate constants of SO_4_^•−^ span 1–2 orders of magnitude more than those of ^•^OH, and the equivalent percentiles correspond to lower values of the reaction rate constants in the case of SO_4_^•−^. Therefore, SO_4_^•−^ is both less reactive and more selective than ^•^OH, which means that in the case of SO_4_^•−^, it would be easier to find a contaminant or a group of contaminants having high reaction rate constant(s) with the radical transient, in a framework of interfering agents showing (on average) much lesser reactivity. The presence of interfering agents may thus have lesser impact on SO_4_^•−^-based compared to ^•^OH-based treatments [45], for which the reaction rate constants have the tendency to be more similar for target contaminants and interfering agents.

**Figure 2:**
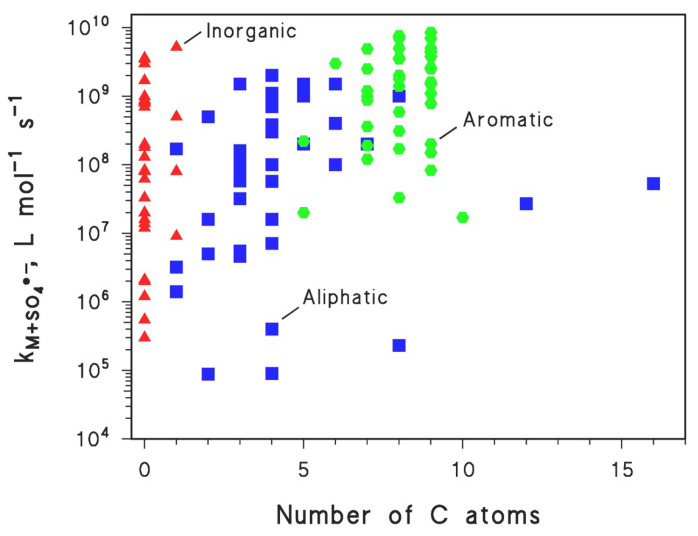
Second-order reaction rate constants ($k_{M+SO_{4}^{•-}}$) of inorganic (▲), aliphatic (■) and aromatic (⬣) compounds M with SO_4_^•−^, as a function of the number of carbon atoms. Rate constant data were derived from the work in [44].

It is also interesting to highlight the reason why two radicals with similar reduction potentials (E° ~ 2.6 V in both cases) [43,44] show different reactivity. The point is that SO_4_^•^^−^ reacts exclusively or almost exclusively via electron transfer, which is easier and faster in the case of heteroatoms (S, N) present on aromatic or aliphatic compounds, compared to inorganic species. This issue is depicted in Figure 2, showing that SO_4_^•−^ is usually more reactive towards aromatics. Compared to SO_4_^•−^, ^•^OH can be involved in a wider range of reactions that, apart from electron abstraction, also include H-atom abstraction and addition to double bonds and aromatic rings [44,52]. Therefore, ^•^OH is still able to react fast with compounds, for which the reactivity with SO_4_^•−^ is rather poor. On this basis, the oxidative removal of many aromatic contaminants with SO_4_^•−^ may provide a favorable scenario, where the contaminants themselves react fast with SO_4_^•−^ and compete favorably with the interfering agents. Indeed, interfering agents usually include an important fraction of aliphatic as well as inorganic compounds.

The above discussion accounts for the current interest in the development of SO_4_^•−^-based advanced oxidation processes, as an alternative or as a complement to the ^•^OH-based ones. Unfortunately, however, it is very difficult to devise a treatment technique that is based exclusively on SO_4_^•−^. A first reason is that ^•^OH and SO_4_^•−^ have very similar standard reduction potentials. Moreover, the reduction of ^•^OH is strongly enhanced in acidic conditions, while that of SO_4_^•−^ is much less dependent on pH: as a consequence, there is a wide pH range where SO_4_^•−^ is able to oxidize water and OH^−^ to ^•^OH, or to its conjugate base O^•−^.

Therefore, even if a treatment technique is initially designed to produce SO_4_^•−^ as the only reactive species, in most cases the system will contain a mix of ^•^OH and SO_4_^•−^. To explain this, consider the thermodynamics of systems containing H_2_O + SO_4_^2−^ + ^•^OH + SO_4_^•−^, which can be described by the following semi-reactions and equilibria [53]:

SO_4_^•−^ + e^−^ → SO_4_^2^^−^ (1)

^•^OH + H^+^ + e^−^ → H_2_O (2)

^•^OH ⇆ O^•−^ + H^+^ (3)

HSO_4_^−^ ⇆ H^+^ + SO_4_^2^^−^ (4)

**Figure 3:**
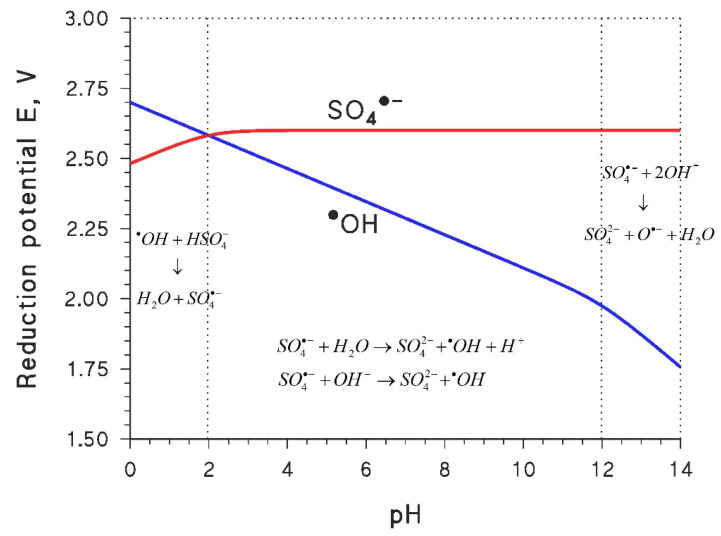
Trends of the reduction potentials of SO_4_^•−^ and ^•^OH, as a function of pH and with standard hydrogen electrode (SHE) as reference electrode. The highlighted pH values correspond to the pK_a_’s of HSO_4_^−^ (~2) and ^•^OH (~12). The thermodynamically favored reactions involving SO_4_^•−^ and ^•^OH are depicted in the relevant pH ranges.

By examining reactions (1)–(4) together, one gets the results shown in Figure 3 as the trends of the reduction potentials of ^•^OH and SO_4_^•−^ as a function of pH. The reported data suggest that a “pure” SO_4_^•−^-based system could be obtained only at pH < 2, where SO_4_^•−^ is unable to oxidize water and, on the contrary, one might have the oxidation of HSO_4_^−^ by ^•^OH (which does not apply, however, if one generates SO_4_^•−^ from the very start).

^•^OH + HSO_4_^−^ → H_2_O + SO_4_^•−^ [k = 1.7 × 10^6^ L mol^−1^ s^−1^] (5)

The kinetics of reaction (5) [43] is consistent with the thermodynamic insight that is reported in Figure 3. In contrast, at pH > 2, which would encompass most conditions found in water treatment, it would be quite difficult to form SO_4_^•−^ without also producing ^•^OH (or O^•−^) as a side process. Indeed, in such conditions one gets that SO_4_^•−^ is a stronger oxidant than ^•^OH/O^•−^, which means that SO_4_^•−^ would be able to oxidize H_2_O/OH^−^ to ^•^OH/O^•−^.

Of course, apart from thermodynamics, the process kinetics is also very important, and the oxidation of H_2_O by SO_4_^•−^ would be in competition with the scavenging of SO_4_^•−^ itself by other dissolved compounds, which depends by dissolved compound concentration and second-order reaction rate constants with SO_4_^•−^. Moreover, because SO_4_^•−^ and ^•^OH often coexist in the same solution, the occurrence and involvement of SO_4_^•−^ and/or ^•^OH in substrate transformation must be assessed on a case-by-case basis. This has often been done in the case of pollutant degradation by ZVI + persulfate, and the studies that have addressed the relative roles of SO_4_^•−^ and ^•^OH will be highlighted whenever relevant in the next section.

## 3. Degradation of Pollutants by ZVI-Fenton/Persulfate

The redox reactivity of ZVI is very interesting because it can be widely modulated depending on the operational conditions. Although it is an electron donor, ZVI induces oxidant activity in the presence of water, dissolved oxygen, and, most frequently, H_2_O_2_, in which circumstances it can trigger the formation of the strong oxidant ^•^OH [5,10]. By exploiting the reductive and/or oxidative capabilities of ZVI alone, it has been possible to use it for the removal of anions [54,55] as a reductant, but also for the treatment of further contaminants such as heavy metals [56,57], dyes [58], and halogenated organic compounds [5,56], where the reducing and oxidizing characters of ZVI have been exploited. The removal of pollutants by ZVI is neither a purely physical adsorption process, nor a purely chemical/electrochemical process. It includes a complex mixture of different pathways like dissolution, adsorption, redox reaction, and precipitation, which happen simultaneously or in a number of steps on the iron surface.

In particular, the mixture of ZVI with persulfate is able to trigger a series of reactions (6)–(10) with generation of reactive radicals (^•^OH, SO_4_^•−^), which can be extensively exploited for the decontamination of wastewater [59,60].

Fe^0^ + 2H_2_O → Fe^2+^ + 2OH^−^ + H_2_ (6)

Fe^2+^ + S_2_O_8_^2−^ → Fe^3+^ + SO_4_^2−^ + SO_4_^•−^ (7)

Fe^0^ + 2Fe^3+^ → 3Fe^2+^ (8)

Fe^2+^*+* H_2_O_2_ → Fe^3+^*+*^•^OH *+* OH^−^ (9)

SO_4_^•^^−^ + H_2_O → SO_4_^2−^ + ^•^OH *+* H^+^ (10)

Note that reactions (7) and (9) describe the Fenton and Fenton-like production of oxidizing species, the interconversion of which is depicted by reaction (10). Moreover, the reactions of iron species with dissolved molecular oxygen (here not shown) can produce Fenton reagents as well. The cycling of Fe species having different redox states is shown by reactions (6)–(9).

Nanoscale ZVI (nZVI) is often used as Fe^0^ form in these processes [30,59], because the favorable surface-to-volume ratio of ZVI nanoparticles enhances their reactivity [22]. The other side of the coin is the fact that nZVI undergoes fast surface oxidation that may hamper its reuse [61]. However, at least in the case of ZVI/H_2_O_2_, it has been found that passivated nZVI (i.e., nZVI covered with a layer of Fe oxides) retains significant Fenton reactivity that would be an advantage in the case of possible reuse [62]. Furthermore, again in the framework of ZVI/H_2_O_2_ it has been shown that the nZVI doses needed to degrade contaminants are quite low and do not affect much the process economics, even in the case of difficult reuse [63]. It is clear that the above findings reported for ZVI/H_2_O_2_ need confirmation in the case of ZVI/PS. Moreover, a family of promising composite materials based on amorphous Fe (metallic glasses) has also been developed recently, with the ability to activate H_2_O_2_, PS, and SO_5_^2−^ and with an interesting performance as far as reuse is concerned [64,65,66,67].

To make some examples of the application of ZVI/PS to decontamination processes, Deng et al. [68] have chosen acetaminophen (APAP) as representative pollutant for pharmaceutical-industry wastewater, applying the iron/persulfate (Fe^0^/PS) Fenton-like process to APAP degradation. They studied the effects of pH, iron dosage, and addition of chelating agents over the Fe^0^/PS system. The highest efficiency (~93%) was achieved with a 1:1 molar ratio between iron and PS. Effective degradation (>90%) was observed in a broad pH range (3–8.5), while the presence of Fe^0^ was assumed to be important for the regeneration of Fe^2+^ by reaction (8) with Fe^3+^. Both SO_4_^•−^ and ^•^OH were found to be involved in APAP degradation. Herein, the production and interconversion of radicals have been observed. Zhang et al. [59] have synthesized nanosized ZVI (nZVI) to carry out nZVI/PS degradation of Norfloxacin (NOR). The highest degradation efficiency (93.8%) of 100 mg L^−1^ NOR was achieved with 100 mg L^−1^ nZVI, 12 mM PS and pH 7.0. The reaction followed the pseudo-first-order kinetic model. From both quenching experiments and EPR analysis it was derived that both SO_4_^•−^ and ^•^OH were involved in NOR degradation, but ^•^OH played the main role. It was found an optimum nZVI dosage for degradation [59], above which it can be assumed that nZVI would scavenge ^•^OH to a significant extent [69]. Moreover, high temperature and PS concentration favored the degradation process. Jiang et al. suggested that Fe^0^ is an efficient source of Fe^2+^ to activate persulfate (S_2_O_8_^2−^) to SO_4_^•−^ for the degradation of bisphenol A (BPA). High initial persulfate or Fe^0^ concentration decreased the BPA removal, but the degradation efficiency increased from 49 to 97% with sequential additions [70]. The use of sequential additions of reagents, which are then consumed in the Fenton reaction, is useful to prevent the reagents to reach excessive concentration values at any time point, differently from a large, single initial addition. Indeed, excess reagents may be detrimental to degradation because they scavenge reactive species (^•^OH and/or SO_4_^•−^) [69]. The pH where the system works at its best efficiency is pH 3, but maintaining this pH value is problematic due to the high cost of the needed reagents. 

It is quite clear from the above discussion that pH and reagent dosage have significant effects over the degradation efficiency. The following section describes the effect of pH over the ZVI/PS process, including the formation of different reactive radicals. 

### 3.1. Effect of pH

Apart from the intrinsic properties of iron, some other operational parameters affect the performance of ZVI-Fenton, including pH, iron dosage, dissolved oxygen, iron pretreatment, temperature, and PS/Fe^0^ ratio [5,50,71]. Among all of the above factors, pH plays a crucial role because it affects both the rate of generation of radicals and the speciation of many contaminants, thereby highly affecting the degradation performance.

Wu et al. [72] have observed higher degradation of sulfamethazine in acidic compared to basic media. In particular, in the presence of PS the degradation efficiency decreased from 91% to 34% as the initial pH was increased from 5 to 10. Degradation was significantly improved (95–98%) in the whole pH range upon addition of H_2_O_2_, but it was still slightly higher at pH 5. Overall, these data suggest that acidic pH may be favorable to the formation of oxidizing species such as ^•^OH and SO_4_^•−^.

As similar decrease in degradation efficiency with increasing pH (3–11) has been reported by Zhang et al. [73] who used ultrasound-nZVI/PS to degrade chloramphenicol (CAP). The effect of pH was attributed to several factors, including (i) precipitation of Fe at alkaline pH and passivation of the ZVI surface, which gets covered by a layer of Fe (hydr)oxides that hinder CAP decomposition; (ii) lower dissolution of ZVI at higher pH, which reduces the availability of Fe^2+^ that is required to activate PS; and (iii) scavenging of SO_4_^•−^ by H_2_O and hydroxyl ions at basic pH. However, the replacement of SO_4_^•−^ by ^•^OH as reactive species is not always detrimental to degradation: the effect depends on substrate, operational conditions, and the possible occurrence of interfering agents, thus it cannot be generalized to all circumstances. Overall, SO_4_^•−^ was shown to play a more important role than ^•^OH in the degradation of CAP in the studied system [73]. The elevated performance of ZVI/PS at acidic pH explains why Diao et al. used this technique for the treatment of acid mine drainage water, to achieve the removal of atrazine (ATZ) [30]. Maximum ATZ removal (84%) was observed at pH 4 and both radicals (SO_4_^•−^, ^•^OH) were involved, with a dominant role played by SO_4_^•−^. Du et al. have carried out degradation of Acid orange 7 (AO7) with granular red mud reinforced by ZVI and persulfate (Fe@GRM/PS) [74]. The study covered a very broad pH range (1–13), and the degradation efficiency was consistently decreased from 94% down to 18% as the pH increased. There is also evidence that adsorption of AO7 on the surface of Fe@GRM played an important role in the degradation process.

Different quenching chemicals have been used to mask the effect of radicals or to capture them. For instance, 5,5-dimethyl-1-pyrroline N-oxide (DMPO) was utilized to capture SO_4_^•−^ and ^•^OH in case of fenitrothion [75], methanol for sulfadiazine [76], NaNO_2_ for acetaminophen [68], and ethanol for diuron [77].

In summary, it is indicated from the above discussion that the process triggered by ZVI/PS has an important dependence on pH. Results from the above and additional studies are reported in Table 1, which highlights both the effect of pH and the role of the reactive radical species (SO_4_^•−^ and/or ^•^OH) in substrate degradation. The table shows that acidic conditions are favorable to the Fenton-like degradation in the vast majority of cases, with only few exceptions. Precipitation of Fe(III) (hydr)oxides with increasing pH, and enhanced dissolution of ZVI with higher occurrence of Fe^2+^ in acidic conditions, are the most likely general explanations for the observed pH effect.

Another issue that is linked to pH is the fact that oxidation of H_2_O/OH^−^ to ^•^OH by SO_4_^•^^−^ is favored in neutral to basic solutions as compared to acidic ones, as already discussed in Section 2. The choice of using PS over H_2_O_2_ as Fenton oxidant is usually motivated by the desire to produce SO_4_^•^^−^ instead of ^•^OH as reactive species (e.g., because of higher SO_4_^•^^−^ selectivity). Therefore, a further argument in favor of operation at acidic pH is represented by the possibility to obtain a simpler system with only one (or a strongly prevailing) reactive species, SO_4_^•−^. Indeed, whenever allowed in the framework of water treatment, the operation of ZVI/PS at acidic pH would combine higher Fenton-like reactivity with higher selectivity due to SO_4_^•^^−^.

### 3.2. Effect of Inorganic Anions

#### 3.2.1. Chloride

The effect of chloride ions on the ZVI/PS process has been controversial because chloride can induce some contrasting processes in Fenton-like systems. In particular, Cl^−^ can boost the corrosion of Fe^0^ [87], and it can also scavenge SO_4_^•−^ and/or ^•^OH with generation of reactive chlorine species (RCS) such as Cl_2_^•−^, Cl^•^, and Cl_2_ [88]. Note that scavenging of SO_4_^•−^ by Cl^−^ can take place at any pH value, while net scavenging of ^•^OH by Cl^−^ occurs only at acidic pH [43,44]. Depending on the conditions, the overall effect of chloride can be either an enhancement or an inhibition of pollutant degradation. To account for inhibition, it has been invoked the scavenging by chloride of strong oxidizing species (SO_4_^•−^, ^•^OH) that are replaced by less reactive RCS such as Cl_2_^•−^ [89].

SO_4_^•−^ + Cl^−^ → Cl^•^ + SO_4_^2−^  k = 2.7 × 10^8^ M^−1^ s^−1^ (11)

Cl^•^ + Cl^−^ → Cl_2_^•−^    k = 4.4 × 10^8^ M^−1^ s^−1^ (12)

Cl^•^ + H_2_O → HOCl^•−^ + H^+^  k = 2.5 × 10^5^ s^−1^ (13)

Cl_2_^•−^ + Cl_2_^•−^ → Cl_2_ + 2Cl^−^   k = 2.1 × 10^9^ M^−1^ s^−1^ (14)

HOCl^•−^ → ^•^OH + Cl^−^   k = 6.1 × 10^9^ s^−1^ (15)

Interestingly, there is evidence that the Fenton-like systems based on ZVI/PS have an improvement in performance in the presence of chloride when the concentration of the latter is low (around 1 mM), while degradation worsens considerably at high chloride (≥10 mM). A reasonable explanation is that Cl^−^ at low concentration activates ZVI corrosion and stimulates the activation of PS by Fe^2+^ [21]. On the other hand, elevated Cl^−^ concentration would rather produce scavenging of SO_4_^•−^ (and/or ^•^OH) [90], replaced by less reactive RCS (Cl_2_^•−^, Cl^•^, Cl_2_).

Coherently, Kim et al. have reported that different concentrations of chloride have different effects on ZVI/PS [89]. They concluded that there is an optimum value of [Cl^−^] for which degradation is most efficient (170 mM Cl^−^, equivalent to ~1% dissolved salts that is in the range of brackish waters). Indeed, based on the obtained results, it was inferred that pollutant degradation by ZVI/PS might be more efficient in brackish waters than in either freshwater, or more saline waters (600 mM Cl^−^, equivalent to ~3.5% dissolved salts as found in seawater).

Chlorinated by-products can be formed during the process and they may be toxic. Although this issue has not been explored much so far, a few intermediate chlorinated by-products have been detected as reported here in Table 2. The formation of chlorinated by-products is quite likely in the presence of RCS and of electron-rich substrates [91]. Indeed, SO_4_^•−^ can oxidize Cl^−^ to produce the chlorinating agents Cl^•^, Cl_2_ upon radical condensation, and Cl_2_^•−^ upon reaction between Cl^•^ and Cl^−^ [44]. The table also reports whether Cl^−^ enhanced or suppressed the degradation of pollutants at the chloride concentrations used in each relevant study.

#### 3.2.2. Nitrate/Nitrite

The effect of nitrate and nitrite ions over Fenton systems based on hydroxyl and sulfate radicals has not been explored much. However, in the presence of nitrate and especially nitrite, one can expect scavenging of sulfate radicals as shown in equations (16,17), as well as of ^•^OH.

NO_3_^–^ + SO_4_^•−^ → NO_3_^•^ + SO_4_^2^^−^ (16)

NO_2_^–^ + SO_4_^•−^ → NO_2_^•^ + SO_4_^2^^−^ (17)

The scavenging process is expected to produce the replacement of SO_4_^•−^ (and ^•^OH) with less reactive radicals (NO_3_^•^ and especially NO_2_^•^), which should inhibit Fenton degradation, but the actual outcome depends on the conditions and the target substrate. The nitrate radical would regenerate nitrate by reacting, thus nitrate is not expected to change in concentration due to this process. Conversely, nitrite is oxidized to nitrate.

For instance, Guo et al. have recently reported the effect of nitrate on the decontamination of sulfadiazine by ZVI/PS [102]. For reasons that are still to be explained, a small amount of nitrate (<10 mM) was found to enhance the removal efficiency. In contrast, the removal of sulfadiazine was inhibited in the presence of higher nitrate concentrations (10–50 mM). The latter effect was attributed to a scavenging process (reaction (16)), combined to the lower reactivity of NO_3_^•^ when compared with SO_4_^•−^ [44,53].

Inhibition of pollutant degradation by PS/SO_4_^•−^ has also been observed in the presence of nitrite at relatively high concentration values (>100 µM) [96]. The oxidation of NO_2_^−^ to NO_2_^•^ by SO_4_^•−^ (reaction 17) is expected to induce a double effect: (i) inhibition of pollutant degradation, because NO_2_^•^ is considerably less reactive than SO_4_^•−^ (and than NO_3_^•^ as well: indeed, nitrite has the potential to inhibit degradation at a higher extent than nitrate if concentrations are comparable) [44,53], and (ii) formation of nitrated by-products, because of the activity of NO_2_^•^ as nitrating agent. Coherently, the degradation of 2-chlorophenol in the presence of SO_4_^•−^ and nitrite has been reported to form different chloronitrophenols, including 2C4NP and 2C6NP [96]. Because these nitro-derivatives that are formed in the process may be toxic, their production should be considered and avoided whenever possible.

To date, only a minority of studies that investigated the effect of nitrate/nitrite on SO_4_^•−^-induced degradation has focused on the formation of nitrated by-products. A short summary of the investigated pollutants, the influence of nitrate/nitrite on their degradation and the detected by-products is given in Table 3.

#### 3.2.3. Carbonate/Bicarbonate

Both HCO_3_^−^ and CO_3_^2−^ can scavenge SO_4_^•−^ (as well as ^•^OH) and finally produce the carbonate radical, CO_3_^•−^ reactions (18) and (19). Although CO_3_^•−^ reacts mainly through electron/hydrogen transfer in a similar way as SO_4_^•−^, CO_3_^•−^ is considerably less reactive than SO_4_^•−^ because of the lower one-electron reduction potential [44,53]. Because of reactions (18) and (19), one might expect that carbonate and bicarbonate inhibit the degradation of pollutants in the presence of ZVI/PS. This is often observed, but in several circumstances the scenario is more complex because the effects of carbonate and bicarbonate on degradation also depend on their concentration, the pH, and the nature of target pollutant(s).

SO_4_^•−^ + CO_3_^2−^ → CO_3_^•−^ + SO_4_^2−^ (18)

SO_4_^•−^ + HCO_3_^−^ → CO_3_^•−^ + H^+^ + SO_4_^2−^ (19)

For instance, Bennedsen et al. have investigated the influence of carbonate over PS activation for the degradation of *p*-nitrosodimethylaniline as model pollutant [99]. Notably, in that case the role of CO_3_^2−^ could not be assimilated to that of a mere scavenger. A similar, positive effect of HCO_3_^−^/CO_3_^2−^ on pollutant degradation by PS activation has been reported by Hayat et al. too [85].

Zhao et al. have studied the effect of water matrices including natural organic matter (NOM) and bicarbonates over the degradation of 2-chlorophenol by PS activation. They found that bicarbonate inhibits degradation due to SO_4_^•−^ scavenging. However, it should also be considered that bicarbonate acts as a buffer for the reaction solutions [96]. Similarly, negative effects of CO_3_^2−^/HCO_3_^−^ on degradation have been reported in the cases of propranolol and sulfamethoxazole. A short summary of the influence of carbonate/bicarbonate over oxidation by SO_4_^•−^ is provided in Table 4, which also highlights their positive/negative behavior along with the target pollutant under study.

As a final remark, it appears that the effect of HCO_3_^−^ and CO_3_^2−^ over the degradation of pollutants by SO_4_^•−^ needs additional investigation, because it is likely to depend on target pollutant, reaction conditions and other parameters.

## 4. Conclusions

The Fenton-like process based on ZVI and persulfate (ZVI/PS) has the potential to overcome the drawbacks of both homogeneous Fenton (production of sludge, very narrow operational pH interval) and the ^•^OH-based advanced oxidation processes (non-selective behavior of the hydroxyl radical). However, it is important to consider that it is difficult to obtain a pure SO_4_^•−^-based oxidation, because of the ability of SO_4_^•−^ itself to oxidize water and OH^−^ to ^•^OH at pH > 2. Therefore, in most cases one will get a mixed process where both ^•^OH and SO_4_^•−^ take part at different degrees in pollutant transformation.

The operational pH value plays an important role in the degradation efficiency of pollutants by ZVI/PS. Usually the degradation is most efficient at acidic pH, for two main reasons: (i) precipitation of Fe(III) as the pH increases, which hampers the recycling of Fe species and decreases, as a consequence, the production of reactive radicals (SO_4_^•−^ and ^•^OH); (ii) dissolution of ZVI at acidic pH, which provides more Fe species (and especially Fe^2+^) for the Fenton reaction. Despite these limitations, the useful pH range for degradation by ZVI/PS is usually wider than for homogeneous Fenton. In homogeneous Fenton systems, when Fe^2+^ is completely oxidized to Fe(III) by H_2_O_2_ or PS, and Fe(III) precipitates, reactivity is totally suppressed. In contrast, ZVI might still act as a source of reactive Fe species even at relatively high pH values, despite a non-negligible loss in reactivity.

Inorganic anions that usually occur in surface waters have the potential to act as scavengers of SO_4_^•−^ and/or ^•^OH, producing less reactive radical species and inhibiting degradation as a consequence. However, in particular conditions and with some anions (Cl^−^, HCO_3_^−^, CO_3_^2−^) one might observe enhanced degradation. Therefore, compared to expectations based on radical scavenging, the degradation of pollutants by ZVI/PS might become peculiarly fast in some saline waters. In such cases one should take into account the possible formation of harmful by-products, however. For instance, reactive chlorine species (Cl_2_^•−^, Cl^•^, and Cl_2_, produced by chloride oxidation) have the potential to form chlorinated compounds, while NO_2_^•^ (produced by nitrite oxidation) acts as nitrating agent. The composition of wastewater may be very variable; thus, it is difficult to figure out which anion’s effect may be the most significant. However, it appears that nitrite affects degradations by ZVI/PS at quite high concentration values, which would be found in water matrices in only a minority of cases. The tested concentrations of the other ions (NO_3_^−^, Cl^−^, HCO_3_^−^, and CO_3_^2−^) are quite comparable with typical water levels, thus the prevailing effect would largely depend on the particular composition of the matrix under treatment. In the case of saline waters, it can be predicted that the effect of chloride would usually be very important. Overall, it can be concluded that the use of the ZVI/PS system can be very beneficial to remove aromatic pollutants easily in the presence of interferents such as aliphatic compounds and inorganic ions.

## Figures and Tables

**Table 1:** Summary of the effect of pH along with generation of respective radicals with different pollutants. Note that Psi = porous silicate.

Pollutants	Experimental Conditions	pH (% Degradation)	Main Reactive Species	Reference
Acetaminophen	Pollutant concentration: 0.066 mM ZVI: 0.1–1 g/L PS: 0.4 mM	3–8.5 (>90%) 1.5 (49.51%) 10.0 (16.57%)	SO_4_^•−^, ^•^OH	[68]
Acid Orange 7	Pollutant concentration: 200 mg^−1^ g Iron source: 0.1–1.4 g/L PS: 5–500 mM	1.0 (93.95%) 3.0 (93.22%) 5.0 (91.34%) 7.0 (66.25%) 9.0 (52.52%) 11.0 (21.62%) 13.0 (18.26%)	SO_4_^•−^	[74]
Anisole	Pollutant concentration: 1 mM PS: 0.25–0.5 M	11.0 (40%) 12.0 (>99%)	SO_4_^•−^, ^•^OH	[47]
Arsenic	Pollutant concentration: 50 µM ZVI:0.1–0.4 g/L PS: 0.5–10 mM	3.0(>99%) 5.0 (98%) 7.0 (96%) 9.0 (60%) 11.0(51%)	SO_4_^•−^, ^•^OH	[36]
Atrazine	Pollutant concentration: 2.5–15 mg/L ZVI: 0.25–1 g/L PS: 0.25–0.50 mM	4.0 (84%)	SO_4_^•−^ (84%), ^•^OH	[30]
Atrazine	Pollutant concentration: 25 mg/L ZVI/BC: 175 mg/L PS: 2 mM	3.0 (83.77%)	SO_4_^•−^, ^•^OH	[78]
Chloramphenicol	Pollutant concentration: 0.05 M ZVI: 0.12–4 mM PS: 0.25–3 mM	3.0 (95.1) 5.0 (94.3) 7.0 (93.4) 8.5 (93.2) 10.0 (92.5)	SO_4_^•−^, ^•^OH (Dominant)	[79]
Diuron (3-(3,4-Dichlorophenyl)-1,1-Dimethylurea)	Pollutant concentration: 0.05 M ZVI: 0.05–1 mM PS: 0.5 M	3.0(82%) 7.0 (65%) 9.0 (No Degradation) 11.0 (No Degradation)	SO_4_^•−^ (Dominant), ^•^OH	[77]
Fenitrothion	Pollutant concentration: 10 mg/L PS:Fe^0^: 1:1.5 molar ratio PS: 0.1M	3.0 5.0 7.0 9.0	SO_4_^•−^	[75]
Naproxen	Pollutant concentration: 25 µM ZVI:0.25–1.50 mM PS: 0.1–0.5 mM	3.0 5.0 7.0 9.0	SO_4_^•−^, ^•^OH	[80]
Nitrobenzene	Pollutant concentration: 1 mM PS: 0.25–0.5 M	11.0 (40%) 12.0 (>60%)	^•^OH	[47]
Nitrobenzene	Pollutant concentration: 200 mg/L ZVI: 0.75 g/L Na_2_S_2_O_8_: 26.8mM	5.0 (100%)	SO_4_^•−^, ^•^OH	[81]
Norfloxacin	Pollutant concentration: 100 mg/L ZVI: 0.075–0.3 g/L PS: 3 mM	3.0 (>90%) 4.5(>90%) 7.0 (93.8%) 9.5 (89.9%) 11.0 (80.8%)	SO_4_^•−^, ^•^OH	[59]
Orange G	Pollutant concentration: 100 mg/L Psi@ZVI: 0.2 g/L PS: 16 mM	3.0 (99.66%) 5.0 (98.34%) 6.9 (98.93%) 8.0 (98.89%) 10.0 (96.76%)	SO_4_^•−^, ^•^OH	[82]
Propranolol	Pollutant concentration: 40 µM ZVI: 0.15 g/L PS: 1 mM	3.0 (97%) 4.5 (94.2%) 7.0 (89.4%) 11.0 (35.4%)	SO_4_^•−^, ^•^OH	[83]
*p*-Chloroaniline	Pollutant concentration: 0.05 mM ZVI: 0.70 g/L PS: 2.5 mM	4.0 (100%) 9.0 (43.59%) 11.0 (41.52%)	SO_4_^•−^, ^•^OH	[84]
1-(6-Chloro-3-Pyridylmethyl) -*N*-Nitro-Imidazolidin-2-Ylideneamine	Pollutant concentration: 30 ppm ZVI: 0.5–3 g/L PS: 2.5–15 mM	7.0 (88%)	SO_4_^•−^, ^•^OH	[85]
Sulfadiazine	Pollutant concentration: 20 mg/L ZVI: 0.92 mM PS: 1.84 mM	3.0–7.0 (95.7–98.4%) 10.0 (35.7%)	SO_4_^•−^	[76]
Reactive Blue 19	Pollutant concentration: 0.3 mM ZVI: 0.8 g/L PS: 10 mM	3.0 (99%) 5.0 7.0 9.0	SO_4_^•−^	[86]

**Table 2:** Effect of chloride ion over pollutant degradation (+ve: Positive effect; −ve: negative effect), and formation of chlorinated by-products.

Pollutant	pH	Effect of Chloride (+ve/−ve/=)	Chlorinated by-Products	Reference
Acid Orange 7	3–11	+ve (1–100 mM) −ve (>100 mM)	5-Chloroisobenzofuran-1,3-Dione, 1-Chloro- 2-(Dimethoxymethyl)Benzene, 1-(3-Chlorophenyl) Propan-1-One 2-Chlorobenzaldehyde	[92]
Bisphenol A	6–8	+ve	Trichloronitromethane	[93]
Carbamazepine	6–8	+ve	Trichloromethane, Trichloroacetonitrile, Trichloronitromethane	[94]
Chloramphenicol	5–8	Absence of Chloride	Dichloroacetamide	[95]
Chloramphenicol	3–9	+ve (<1 mM)		[79]
4-Chlorophenol	2–7	+ve (1 mM) −ve (>5 mM)		[21]
2-Chlorophenol	7.9	Absence of Chloride	2-Chloro-4- Nitrophenol (2C4NP), 2-Chloro-6-Nitrophenol (2C6NP)	[96]
(2-Chloro-*N*-2,6-Diethylphenyl- *N*-(Methoxymethyl)Acetamide	7.61–8.76	+ve	Trichloromethane, 1,1,3-Trichloro-2-Propanone 1,3-Dichloro-2-Propanone	[97]
2,4-Di-Tert-Butylphenol	7–8	+ve	Trichloromethane	[98]
Perchloroethylene	7	No Effect up to 28 mM		[99]
Propanolol		−ve (≥5 mM)		[83]
Phenol	2.5	+ve (25–200 mM) −ve (>400 mM)		[89]
Phenol	3.9–4.4	No Effect		[100]
Rhodamine B	2–12	+ve up to 50 mM Cl^−^		[101]
Sulfadiazine	4.0	+ve up to ≤10 mM		[102]
Sulfamethoxazole		+ve		[103]

**Table 3:** Effect of nitrite/nitrate ion over pollutant degradation.

Pollutant	pH	Effect of Nitrate/Nitrite	Nitro-Derivatives as Byproducts	Reference
Bisphenol A	7.0	NO_2_^−^ +ve	Trichloronitromethane	[93]
Chloramphenicol	3.12–5.4	NO_3_^−^/NO_2_^−^ −ve/−ve		[79]
2-Chlorophenol	7.0	NO_3_^−^ +ve (50–100 µM) −ve (>100 µM)	2-chloro-4- nitrophenol (2C4NP), 2-chloro-6-nitrophenol (2C6NP)	[96]
(2-Chloro-*N*-2,6-Diethylphenyl- *N*-(Methoxymethyl)Acetamide		No effect		[97]
Propanolol		NO_3_^−^ −ve (≥5 mM)		[83]
Phenol	3.9–4.4	No effect		[100]
Sulfadiazine	4.0	NO_3_^−^ (+ve < 10 mM) −ve (10–50 mM)		[102]
Sulfamethoxazole		NO_3_^−^ +ve		[103]

**Table 4:** Effect of carbonates/bicarbonates.

Pollutant	pH	Effect of Bi/Carbonate	Reference
2-Chlorophenol	7.9	+ve	[96]
Imidacloprid	7.0	+ve	[85]
Phenol	7.4–11.3	+ve	[100]
Propranolol		−ve	[83]
*p*-Nitrosodimethylaniline	12.4	+ve (10–100 mM) in alkaline media −ve in acidic media	[99]
Sulfamethoxazole		−ve	[103]
Tetrabromobisphenol A	7.0–8.5	−ve	[104]
